# Screening for pickiness – a validation study

**DOI:** 10.1186/s12966-016-0458-7

**Published:** 2017-01-07

**Authors:** Silje Steinsbekk, Trude Hamre Sveen, Alison Fildes, Clare Llewellyn, Lars Wichstrøm

**Affiliations:** 1 Department of Psychology, Norwegian University of Science and Technology (NTNU) Social Science, Dragvoll, 7491 Trondheim, Norway; 2 School of Psychology, University of Leeds, Leeds, LS2 9JT UK; 3 Health Behaviour Research Centre, Department of Epidemiology and Public Health, University College London, 1-19 Torrington Place, London, WC1E 6BT UK

**Keywords:** Picky eating, Fussiness, Validation, Questionnaire, Screening

## Abstract

Picky eating is prevalent in childhood and is associated with negative health outcomes. Therefore early detection of pickiness is pertinent. Because no psychometric measure of picky/fussy eating has been validated, we aimed to examine the screening efficiency of the 6-item ‘Food Fussiness’ (FF) scale from the Children’s Eating Behavior Questionnaire using structured psychiatric interviews (the Preschool Age Psychiatric Interview), providing meaningful cut-off values based on a large, representative sample of Norwegian 6 year olds (*n* = 752). Screening efficiency was evaluated using receiver operating characteristic curve analysis, revealing excellent discrimination. The cut-point maximizing the sum of sensitivity and specificity for the scale was found at a score of 3.33 for severe cases and 3.00 when both moderate and severe pickiness were included. The results suggest that the FF scale may provide a tool for identification of clinically significant picky eating, although further assessment may be needed to separate moderate from severe cases.

## Background

Pickiness is the unwillingness to eat specific foods or try new foods, thus limiting dietary variety [1]. Although no uniform definition of pickiness exists, most definitions include both food neophobia (i.e. the unwillingness to try new foods) and the tendency to be highly selective about food [2]. These two aspects of picky eating are highly correlated and have been found to share a common etiology in childhood [3]. We therefore align with a recent review which defined pickiness as ‘the unwillingness to eat familiar foods or try new foods, severe enough to interfere with daily routes to an extent that is problematic to the parent, child, or parent-child relationship [2] (p. 352). Research indicates that 5.6 to 59.3% of young children display picky eating, the rates depending on the definition and assessment methods used [2]. While pickiness typically emerges in early childhood, a substantial number of children continue to display pickiness into school age [4], whereas others become picky eaters after starting school [5]. Pickiness has been associated with lower fruit and vegetable intake and essential nutrient deficiency [1], risk of underweight [6], as well as concurrent and prospective symptoms of psychopathology [7]. Given these health consequences, interventions might be warranted and early detection of pickiness is pertinent. Reviews of childhood pickiness [1, 2] note differences in assessment of picky eating. According to Taylor et al. [2] the methods applied fall into two categories: (1) The use of one or more items from existing questionnaires; and (2) The use of one or more items developed for the specific study. The measures used to assess pickiness range from simple single items (e.g., ‘Is your child a picky eater’ [4]) to more complex questions (e.g. ‘My child is interested in tasting foods s/he hasn’t tasted before’[8]) from multi-item scales (typically 2 to 6 items) [2]. Taylor et al. [2] conclude that validation of more reliable measures of pickiness are needed [1]. To be considered valid the instrument in question must display acceptable levels of accuracy, which can be ensured by comparisons to ‘gold standard’ tests or psychiatric interviews [9]. However, to the best of our knowledge, no earlier study has examined screening for picky eating. The aim of the present study was therefore to examine the screening efficiency of the Children’s Eating Behavior Questionnaire (CEBQ) ‘Food Fussiness’ (FF) scale [8] using a structured psychiatric interview (the Preschool Age Psychiatric Interview (PAPA)) [10], and provide cut-off values based on a large, representative sample of Norwegian children.

## Methods

### Participants and procedure

Families with children born in 2003 and 2004 in Trondheim, Norway were recruited at routine health check-ups for 4 year olds (97.2% attendance). Of those invited to participate (*n* = 3,016), 82.2% consented; a subsample (*n* = 1,250) was drawn to participate in the Trondheim Early Secure Study (TESS). Details of the TESS study are described in Wichstrøm et al. [11]. The present inquiry used data from the second wave of collection only, including 752 children (376 boys, 376 girls) with CEBQ FF data (mean age = 6.7 years, SD = .18). Attrition analyses revealed drop-out was not predicted by gender or pickiness assessed by clinical interview. Parents were mainly of Norwegian origin (92.3%) and were comparable to the Norwegian parent population for the parents’ level of education [12]. The majority of informants were mothers (81.1%).

### Measures

Picky eating was assessed at 6 years by: 1) the 6-item FF scale from the CEBQ (5 point Likert response scale) [8], a parent-reported measure of pickiness assessing both neophobia (e.g. ‘My child refuses new foods at first’) and more general ‘fussy’ eating (e.g. ‘My child is difficult to please with meals’) (α = .89); and 2) the PAPA, a semi-structured psychiatric interview [10]. The CEBQ was developed to capture the range of eating styles seen in children, and the inclusion of eating style constructs was based on an 1) evaluation of the existing literature; and 2) interviews with parents about their children’s eating. Fussiness was a common observation and the FF scale constitutes one of eight eating style dimensions captured by the CEBQ [8]. The questionnaire has been validated against behavioral measures of eating [13], and has shown good test-retest reliability [8].

The PAPA interview was administrated by trained personnel holding at least a bachelor’s degree in a relevant field and with substantial practice in working with children and families. Parents were interviewed about their child’s food preferences, appetite, restricted consumption of foods, and resulting impaired functioning. The interviewer decided whether pickiness was present and probed until s/he could categorize the children: 0 = no restricted intake; 1 = moderately restricted intake (child only eats food s/he likes); 2 = severe pickiness (substantial pickiness; separate meals must be made for the child). Nine percent of videotaped recordings were recorded by blinded interviewers, with high interrater reliability (ICC = .92).

Children’s height and weight were measured using digital scales (Heightronic digital stadiometer: QuickMedical,Model 235A and Tanita BC420MA). Weight was measured to the closest .1 kg and correction for light indoor clothing (0.5) was applied. BMI SDS (Body Mass Index Standard Deviation Score) was estimated [14–16]. Parental occupation coded according to the International Classification of Occupations [17] (6-point scale: 1 = Manual workers, 6 = Leaders) was used to measure socioeconomic status.

### Statistical analyses

Descriptive analyses were conducted in SPSS 22, using the Complex Samples option. General Linear Modeling (GLM) was applied to examine the association between the mean FF scale score and BMI SDS.

The screening efficiency of the FF scale was evaluated using non-parametric receiver operating characteristic (ROC) curve analysis, which determines the area under the curve (AUC) for the FF scale against the PAPA. The AUC indicates the probability that a randomly selected subject with PAPA-defined pickiness and a randomly selected subject not categorized as a picky eater according to the PAPA would be correctly distinguished based on their FF scale scores. The sensitivity/specificity pairs generated through the ROC analysis were used to select a threshold for identification of clinical cases (PAPA-defined pickiness). The analysis was performed twice; first using both moderate and severe pickiness as the main outcome (0 = ‘normal’; 1 = ‘moderate/severe’); secondly, using severe cases only (0 = ‘normal/moderate’; 1 = ‘severe’). At a given cut-point, sensitivity shows the proportion of children positively screened with the FF scale among children with PAPA-defined pickiness; specificity denotes the proportion of children with a negative FF scale screen among non-picky eaters. The positive predictive value (PPV) and negative predictive value (NPV) were also calculated; the PPV provides the probability that a child who screens positive on the FF scale does have PAPA-defined pickiness, the NPV provides the probability that a child who screens negative does not have the condition (i.e. is a noncase). ROC analyses were performed in STATA 13. Analyses were performed using probability weights because we oversampled for emotional and behavioral problems at baseline to increase variability and statistical power (for details, see [11]).

## Results

**Fig. 1:**
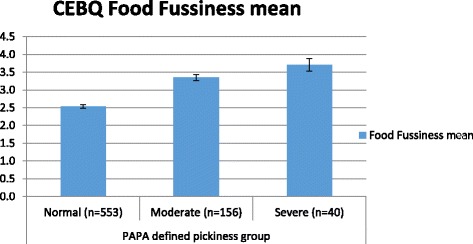
Mean Child Eating Behavior Questionnaire (CEBQ) Food Fussiness scale scores with 95% confidence intervals for each group of pickiness as defined by the Preschool Age Psychiatric Assessment (PAPA)

The mean FF scale score was 2.78 (95% CI: 2.72–2.82). As expected, pickiness was negatively associated with children’s BMI SDS (unstandardized coefficient = −.10; 95% CI = −.15, −.04; *p* ≤ .01), whereas parental socioeconomic status was unrelated to pickiness (unstandardized coefficient = .02, 95% CI = −.03, .06; *p* = .54). Based on the PAPA, 74.2% of children showed no picky eating, 20.9% had moderate, 4.9% displayed severe pickiness. No gender differences were observed. Figure 1 shows the mean FF scale scores for each group of PAPA-defined pickiness (‘No pickiness’: mean FF scale score = 2.54; 95% CI = 2.49, 2.59; ‘Moderate pickiness’: mean FF scale score = 3.35; 95% CI = 3.26, 3.44; ‘Severe pickiness’: mean FF scale score = 3.71; 95% CI = 3.53, 3.89).

The FF scale obtained an AUC value of .87 (.82 −.92) for severe cases and .85 (.82 −.88) for moderate/severe cases, which is excellent discrimination [18]. The cut-point maximizing the sum of sensitivity and specificity for the scale was 3.33 for severe cases, and 3.00 for moderate/severe cases (Table 1).

**Table 1:** Receiver operating characteristics analyses for the children’s eating behavior questionnaire food fussiness scale

CEBQ FF Score	Pickiness moderate/severe (*n* = 196)		Pickiness severe (*n* = 40)	
	Sensitivity	Specificity	Sensitivity	Specificity
2.67	0.925	0.523	0.981	0.427
2.83	0.871	0.643	0.928	0.533
3.00	0.817	0.726	0.928	0.612
3.17	0.715	0.819	0.882	0.710
3.33	0.608	0.884	0. 767	0.783
3.50	0.472	0.923	0. 704	0.848
3.67	0.388	0.942	0.580	0.879

Note: *CEBQ FF score* Children’s Eating Behavior Questionnaire Food Fussiness scale score

At the selected cut-points sensitivity and specificity exceeded 0.70 for both groups. Raising the cut-point led to a decrease in sensitivity, lowering the cut-point compromised specificity. PPV and NPV were 0.16 (0.11–0.22) and 0.98 (0.97–0.99) for severe cases and 0.51 (0.45–0.57) and 0.92 (0.89–0.94) for moderate/severe cases.

## Discussion

The present study is the first to explore the utility of a psychometric measure for screening for pickiness. We assessed the efficiency of the CEBQ FF scale to screen for pickiness defined by clinical interview, and provided cut-off scores. The mean FF scale score was comparable to earlier studies [8] and was negatively associated with children’s BMI SDS as accords former findings [6, 19], which adds to the construct validity of the present work. The FF scale excellently discriminated picky eaters from non-picky eaters, both when moderate cases were included and when they were not (AUCs = .85 and .87, respectively). Because pickiness is associated with negative health outcomes and further assessment may be needed after being screened positive, we prioritized high sensitivity. The majority of cases were detected (approximately 80%) with the suggested cut-offs of 3.00 for moderate and severe cases combined, and 3.33 for severe cases only; and rates of false negatives were low. However, high sensitivity is commonly accompanied with comparatively low PPV [9]: i.e. many children are typically falsely screened as positive. The risk of false positives in the present study was particularly high when only severe pickiness was included. This reflects a tendency seen in community screening; false positives are high when the prevalence of severe cases is low. This suggests the FF scale cannot separate moderate from severe cases reliably, supported by the PPV increase (.16 to .51) when moderate cases are included. For practical purposes, the use of just one cut point for moderate pickiness might therefore be more prudent to minimize the number of false positives.

Because picky eating varies by age [1], our findings cannot be generalized to other age groups, and replication in other cultures is also required. Nonetheless, the CEBQ FF Scale with the cut offs we recommend may provide a useful tool for identifying non-normative picky eating when the time and expense of conducting in-depth interviews are prohibitive.

## Abbreviations

AUC: Area under the curve
CEBQ: Children’s eating behavior questionnaire
FF: Food fussiness
NPV: Negative predictive value
PAPA: Preschool age psychiatric interview
PPV: Positive predictive value
ROC: Non-parametric receiver operating characteristic
